# Characterization of nAChRs in *Nematostella vectensis* supports neuronal and non-neuronal roles in the cnidarian–bilaterian common ancestor

**DOI:** 10.1186/s13227-019-0136-3

**Published:** 2019-11-02

**Authors:** Dylan Z. Faltine-Gonzalez, Michael J. Layden

**Affiliations:** Department of Biological Sciences, Lehigh University, Bethlehem, PA 18015 USA

**Keywords:** Nicotinic acetylcholine receptor, Cholinergic, Cnidaria, *N. vectensis*

## Abstract

**Background:**

Nicotinic and muscarinic acetylcholine receptors likely evolved in the cnidarian–bilaterian common ancestor. Both receptor families are best known for their role at chemical synapses in bilaterian animals, but they also have described roles as non-neuronal signaling receptors within the bilaterians. It is not clear when either of the functions for nicotinic or muscarinic receptors evolved. Previous studies in cnidarians suggest that acetylcholine’s neuronal role existed prior to the cnidarian–bilaterian divergence, but did not address potential non-neuronal functions. To determine the origins of neuronal and non-neuronal functions of nicotinic acetylcholine receptors, we investigated the phylogenetic position of cnidarian acetylcholine receptors, characterized the spatiotemporal expression patterns of nicotinic receptors in *N. vectensis*, and compared pharmacological studies in *N. vectensis* to the previous work in other cnidarians.

**Results:**

Consistent with described activity in other cnidarians, treatment with acetylcholine-induced tentacular contractions in the cnidarian sea anemone *N. vectensis.* Phylogenetic analysis suggests that the *N. vectensis* genome encodes 26 nicotinic (nAChRs) and no muscarinic (mAChRs) acetylcholine receptors and that nAChRs independently radiated in cnidarian and bilaterian linages. The namesake nAChR agonist, nicotine, induced tentacular contractions similar to those observed with acetylcholine, and the nAChR antagonist mecamylamine suppressed tentacular contractions induced by both acetylcholine and nicotine. This indicated that tentacle contractions are in fact mediated by nAChRs. Nicotine also induced the contraction of radial muscles, which contract as part of the peristaltic waves that propagate along the oral–aboral axis of the trunk. Radial contractions and peristaltic waves were suppressed by mecamylamine. The ability of nicotine to mimic acetylcholine responses, and of mecamylamine to suppress acetylcholine and nicotine-induced contractions, supports a neuronal function for acetylcholine in cnidarians. Examination of the spatiotemporal expression of *N. vectensis* nAChRs (*NvnAChRs*) during development and in juvenile polyps identified that *NvnAChRs* are expressed in neurons, muscles, gonads, and large domains known to be consistent with a role in developmental patterning. These patterns are consistent with nAChRs functioning in both a neuronal and non-neuronal capacity in *N. vectensis.*

**Conclusion:**

Our data suggest that nAChR receptors functioned at chemical synapses in *N. vectensis* to regulate tentacle contraction. Similar responses to acetylcholine are well documented in cnidarians, suggesting that the neuronal function represents an ancestral role for nAChRs. Expression patterns of nAChRs are consistent with both neuronal and non-neuronal roles for acetylcholine in cnidarians. Together, these observations suggest that both neuronal and non-neuronal functions for the ancestral nAChRs were present in the cnidarian–bilaterian common ancestor. Thus, both roles described in bilaterian species likely arose at or near the base of nAChR evolution.

## Background

We are interested in the ancestral functions of nicotinic acetylcholine receptors. Nicotinic acetylcholine receptors (nAChRs) are ionotropic ligand-gated ion channels that open in response to acetylcholine binding [1]. Muscarinic acetylcholine receptors (mAChRs) are metabotropic G-protein coupled receptors that induce a range of cellular responses to acetylcholine [2]. The nAChRs and mAChRs have been well characterized in bilaterian species (insects, vertebrates, annelids, echinoderms, hemichordates, mollusks, nematodes, etc.). They are expressed in a wide range of tissues, and are known to function as receptors for cell signaling regulating cell-to-cell contact, proliferation, differentiation, migration, gene expression, and apoptosis in responding cells [2–4]. However, nAChRs and mAChRs are most well known for their role in chemical synapses, where they regulate the excitability of neurons and muscles [2, 5].

Outside of the bilaterians, nAChRs have been widely described in the cnidarians (sea anemones, corals, and *Hydra*) [6–11]. However, nAChRs have not been identified in any of the poriferan, ctenophore, or placozoan genomes [12–17], or in any non-metazoan species. While mAChR are well described in bilaterians, a single mAChR in the *Hydra* genome is the only described cnidarian mAChR [18]. A single receptor with structural similarities to muscarinic receptors was identified in the amoeba *Acanthamoeba* [19]. However, lack of sequence conservation argues that this receptor evolved convergently [19]. Because nAChRs and mAChRs are only found in cnidarians and bilaterians, which are sister taxa [20], the most likely evolutionary scenario is that these receptors evolved once prior to cnidarian–bilaterian divergence. Because investigations of nAChRs and mAChRs have been restricted to bilaterian species, it is necessary to begin to investigate these genes in cnidarians to understand the ancestral and evolved roles for acetylcholine signaling through nicotinic and muscarinic receptors.

Early pharmacological studies hint that nAChRs functioned in neuronal communication and/or neuromuscular junctions in stem cnidarians [21–23]. Dissected oral rings and body strips from the anthozoan cnidarian *Bunodosoma caissarum* contracted in response to either acetylcholine or nicotine [22]. In the hydrozoan *Liriope tetraphylla*, nicotine-induced tentacular contractions, but acetylcholine did not [23]. In hydrozoans, treating *Hydra attenuata* with nicotinic or muscarinic receptor antagonists disrupted contractile burst pulses [21], whereas the treatment of *Hydra piradi* with acetylcholine increased contractile behaviors [24]. While these data indicate that acetylcholine may be a conserved inducer of tissue contraction in cnidarians, early studies lacked reciprocal treatments of agonists, and antagonists in the same species and did not determine if nAChR and/or mAChR receptors are expressed in responding tissues. Recent efforts have shown that acetylcholine signals through chemical synapses to modulate cnidocyte firing in hydrozoans [25], and acetylcholine receptors have been shown to be expressed in cnidocytes in *Hydra vulgaris* [11]. Together, these findings suggest that ancestral cnidarian AChRs likely functioned in neuronal and/or neuromuscular communication.

Whole genome sequences of *Nematostella vectensis*, *Acropora digitifera*, *Clytia hemisphaerica*, and *Hydra* have allowed us to identify, and begin to characterize, the role of acetylcholine receptors with greater detail in cnidarians [7–9, 26]. Recent single-cell RNA-sequencing work in *N. vectensis* has identified expression of nAChRs in neuronal cells, muscle cells, gonads, and the apical tuft of *N. vectensis* larvae [10, 27]. Lacking in the single-cell RNAseq data is the resolution to determine at what stages nAChRs are expressed in each cell type, and the ability to determine how specific nAChR expression is. Single-cell RNA sequencing of *Hydra* polyps identified potential nicotinic acetylcholine receptors within tentacles, nematocytes, neural cells, ectodermal cells, and gland cells, while the muscarinic *Hydra* receptor was only identified in neural tissue and gland cells [11]. The expression pattern of any AChR during *Hydra* development is currently unknown. The conserved expression in neuronal and muscle cells, as well as the pharmacological studies in a number of species, suggests that AChRs functioned in neuronal/neuromuscular communication at the base of the cnidarian lineage.

Here, we characterize the acetylcholine receptors in the anthozoan cnidarian sea anemone *N. vectensis*. *N. vectensis* has 26 nicotinic acetylcholine receptors encoded in its genome. Phylogenetic analysis suggests that the cnidarian receptors radiated independently from bilaterian receptors, which supports the need to independently investigate cnidarian receptors to determine if neuronal and/or non-neuronal functions were present in ancestral nAChRs. In *N. vectensis,* acetylcholine treatment induced tentacle contraction. Treatment with the nAChR agonist, nicotine, mimicked the acetylcholine response, and the nAChR antagonist mecamylamine suppressed both acetylcholine and nicotine mediated tentacular contractions. Interestingly, nicotine also induced contractions of the radial muscles present in endodermal tissue, and mecamylamine suppressed nicotine mediated radial contractions. To gain a broader insight into the potential role(s) of acetylcholine in *Nematostella*, we used mRNA in situ hybridization to determine if nAChRs are expressed in non-neuronal cells. Non-neuronal-like expression in large regions such as ubiquitous expression, endodermal expression, and large domains that encompassed the pharynx and the apical tuft were observed for seven of the 15 genes assessed. Our data suggest that nAChRs likely function in both neuronal and non-neuronal roles in *N. vectensis,* supporting the hypothesis that both functions were present in the ancestral nAChR(s) that gave rise to both the cnidarian and bilaterian receptors.

## Methods

### Phylogenetic analysis

**Fig. 1:**
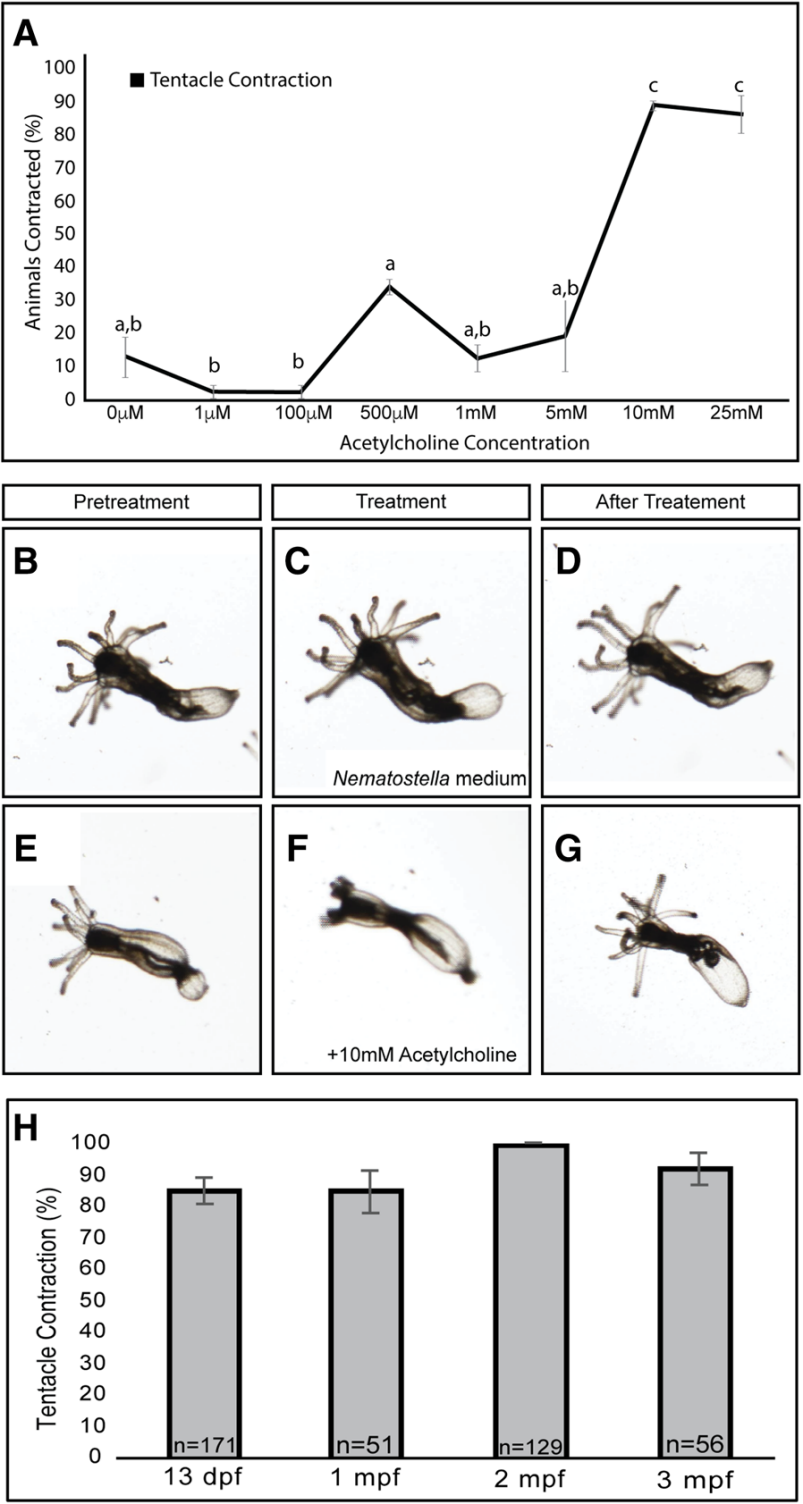
Acetylcholine induces tentacular contractions. **A** Contractile response to 0 µM–25 mM acetylcholine in 3-month-old polyps. The first response to acetylcholine occurred at 500 µM acetylcholine with 33.6 ± 2.4% of animals contracting their tentacles but was not significantly different than the controls (*p* = 0.339). At 1 mM, tentacular contractions occurred in 12 ± 3.97% of polyps tested. At 5 mM, tentacular contractions occurred in 18 ± 10.82% of polyps. Tentacular contractions occurred in 88 ± 1.42% of polyps treated with 10 mM and 85.9 ± 5.63% of polyps treated with 25 mM acetylcholine. Tentacular contractions observed at 10 mM and 25 mM were statistically significant from contractions observed polyps treated with control and lower concentrations of acetylcholine (*p* ≤ 0.05). **B**–**D** Single frame images from movies showing tentacular response to the addition of *Nematostella* growth medium. **E**–**G** Single frame images from movies showing tentacular response to the addition of 10 mM acetylcholine (**F**), and the subsequent relaxation that occurs after treatment (**G**). Response to acetylcholine is not significantly different (*p* = 0.196) between 13 day postfertilization (13 dpf), 2-month post fertilization (2 mpf), or 3-month postfertilization (3 mpf) old polyps (**H**). In all images, oral is to the left. These data were calculated using a one-way ANOVA. **A** Contractile response to 0 µM–25 mM acetylcholine in 3-month-old polyps *F*_*7,18*_= 53.7, *p* < 0.001; (**H**) Response to acetylcholine at different timepoints *F*_*3,8*_= 1.98, *p* = 0.196. Each experiment was performed *N* ≥ 3 times with an *n* ≥ 8/replicate. Points that do not share letters in (**A**) are statistically different from each other. (mpf) month postfertilization. (dpf) day postfertilization

Bilaterian protein sequences were found using keyword searches (e.g., nicotinic acetylcholine receptor, muscarinic acetylcholine receptor, GABAergic receptor, and glycine receptor) to identify sequences within multiple genomic databases (mouse genome informatics, flybase, wormbase, zfin, and NCBI). Non-*N. vectensis* cnidarian protein sequences were collected using bilaterian protein sequences blasted on NCBI or the Clytia hemisphaerica genome browser [26]. *N. vectensis*-coding sequences were collected by blasting bilaterian and cnidarian nicotinic or muscarinic acetylcholine receptor sequences against the *N. vectensis* genome (https://genome.jgi.doe.gov/pages/search-for-genes.jsf?organism=Nemve1), and the NvERTx database [28]. Putative AChRs identified by BLAST hits were then selected and uploaded to ApE v2.0.47, where they were translated. The largest open reading frames identified in the *N. vectensis*-coding sequences were then used for protein alignment [29]. All protein sequences were uploaded into MEGA v7.0.25 software, exported, and then, an MAFFT alignment was performed using the CIPRES gateway [30–32]. We excluded all *N. vectensis* sequences missing characteristic residues required for acetylcholine binding. Phylogenetic analyses were performed using two previously published AChR alignments. The published alignments are unedited protein sequences [33, 34], and an edited alignment that included the double cysteines necessary for acetylcholine binding and the transmembrane domains (T1–T4) [35]. Known bilaterian muscarinic acetylcholine receptors and the single published cnidarian muscarinic acetylcholine receptors were included to determine if any *N. vectensis* sequences cluster within the muscarinic clade. To expand the number of cnidarian muscarinic acetylcholine receptors we also mined the *Clytia hemisphaerica* genome to identify any potential muscarinic acetylcholine receptors [26]. Phylogenies were generated using RaxML and IQ tree with GABA receptors serving as the out-group [6, 34, 36] (Fig. 1; Additional files 1, 2, 3: Figures S1, S2, S3). We determined the LG phylogenetic evolution model using IQ-tree protein Model Finder [37]. The phylogenetic trees were made utilizing IQ tree and RaxML with ultrafast bootstrapping or bootstrapping repeated 1000 times, respectively [38, 39].

### Animal care

Adult *N. vectensis* were maintained at Lehigh University in 17 °C incubators. Culture and spawning of animals were performed according to previously published protocols [40]. Embryos were raised at 22 °C until fixation at the required stage.

### In situ hybridization

Each gene was sub-cloned into pGEM-T (Promega) from mixed stage cDNA using the primers listed in Additional file 4: Table S1. Embryo fixation, RNA probe synthesis, in situ hybridization, and immunohistochemistry protocols were performed as previously described [41, 42]. Juvenile polyps were fixed for 2 h as previously described [43]. When *in situs* returned no pattern, we attempted to remake the probe and repeated the in situ. No expression represents at least two attempts to determine a spatiotemporal pattern.

### Pharmaceutical treatments

All stock concentrations were generated by resuspending pharmaceutical reagents in *Nematostella* medium [1/3X artificial seawater (ASW)]. Reagents used were Acetylcholine hydrochloride (Sigma-Aldrich Cat#A6625), Nicotine hydrogen tartrate salt (Sigma-Aldrich Cat#SML1236), mecamylamine hydrochloride (Sigma-Aldrich Cat.#M9020), and lidocaine (Sigma-Aldrich Cat.#L7757). Concentrations for Acetylcholine, nicotine, and mecamylamine were experimentally determined (see “Results”). Lidocaine was used at a concentration of 5 mM, which was previously shown to be the effective for blocking *N. vectensis* voltage-gated sodium channels [44].

To perform pharmacological treatments, polyps were moved into a concave depression slide for imaging and left to relax until the majority of polyps re-extended their tentacles. Animals that failed to relax after moving were excluded from further analysis. Once the majority of animals relaxed, animals were video recorded for 1 min to assess baseline activity. At the 1 min mark, acetylcholine–chloride or nicotine was added to the depression slide and all contractions were quantified. Because the previous experiments suggested that there is a delay in the effect of some compounds, we continued to monitor animals for an additional 5 min to ensure no additional contractions occurred [23]. Lidocaine and mecamylamine inhibitors were added to animals in *Nematostella* medium 20 min prior to application of nicotine or acetylcholine. The 20 min pretreatment was chosen based on the previous observations in *Nematostella* [44]. Tentacle contractions were measured for each animal and given a score of either complete contraction of all tentacles (1), a complete contraction of ≥ half, but not all of the tentacles (0.5), or no tentacle contraction (0). The total contractions were summed for each treatment and the percentage of contractions per animal was then calculated. We then calculated the mean among the trials. Peristaltic initiation contractions were defined as any inward contraction perpendicular to the oral–aboral axis that occurred within the body column. Peristaltic wave contractions were defined as any propagation of radial contractions along the oral–aboral axis of the body column. Peristaltic initiation contractions and peristaltic wave contractions were then quantified as either being a contraction (1), or no contraction (0). We calculated the percent of animals that contracted per total number of animals tested for each trail. We then calculated the mean for the trials. Controls were performed by adding a volume of *Nematostella* medium equal to the volume of stock pharmaceutical reagent added to the concave imaging slide. Statistical significance was determined using a one-way ANOVA with the Games-Howell post hoc analysis, due to unequal variance, performed in SPSS or a student’s *t* test using Microsoft Excel, when appropriate. Animals used for one experiment were not reused for any subsequent experiments.

### Imaging

Images for in situ hybridization were acquired on a Nikon NTi with a Nikon DS-Ri2 color camera using the Nikon elements software. Time series were performed on a Nikon Eclipse E1000 with a Nikon camera and analyzed on the Nikon elements software. Time series images were analyzed using the Fiji software v.2.0.0-rc-54/1.51 g [45]. All images were edited using Adobe Illustrator and Photoshop and videos were edited using Adobe Premiere Pro (Adobe Inc.).

## Results

### Acetylcholine treatment induces tentacle contractions in *N. vectensis*

We first set out to determine if *N. vectensis* would have responses to acetylcholine similar to those observed in other cnidarians [21–23, 46]. 15% of control animals treated with *Nematostella* growth medium contract their tentacles (Figs. 1A–D, 3H, 4A–C, P; Additional file 5: Video S1). We next tested a dose response of acetylcholine using 1 μM to 25 mM, because this range of concentrations was shown to be effective in other cnidarians [22–24]. We observed no statistically significant increase in contractions between 1 μM and 5 mM (Fig. 1A), but at 10 mM and 25 mM acetylcholine, ≥ 86% of the animals contracted their tentacles (*p* < 0.05) (Fig. 1A, F; Additional files 6, 7: Videos S2, S3). All the remaining experiments were performed with 10 mM acetylcholine. The observed tentacle contractions were typified by a shortening of the proximal–distal axis (Additional file 6: Video S2). This is distinct from tentacle retraction, where tentacles are pulled into the body by contractions around the pharynx. Tentacles relax after contracting (Fig. 1G; Additional files 6, 7: Videos S2, S3), presumably due to degradation of acetylcholine by acetylcholinesterases, which are known to exist and are widely expressed in *N. vectensis* [6, 9, 10, 47]. We did not detect any additional contractions, even after 5 min, and the rate of peristaltic contractile waves that occurred in the body column was unaltered by acetylcholine (Additional file 8: Figure S4A). To confirm that cholinergic modulation of tentacle contractions occurs at all life stages, we treated juvenile, 1-month, 2- month, and 3-month-old polyps with 10 mM acetylcholine. Tentacle contractions were observed in ≥ 84% of animals at all ages, and there was no statistical significance between responses at any age (p > 0.05) (Fig. 1H). This is consistent with our previous observations that juvenile polyp nervous systems resemble adult nervous systems, but have fewer total neurons [41]. We conclude that acetylcholine induces tentacular contractions in *N. vectensis,* which is similar to observed responses in other cnidarians (Reviewed in [46]. However, we cannot rule out that acetylcholine may also regulate other contractions in *N. vectensis.* The musculature responsible for tentacle contractions is derived from the ectoderm [48]. Acetylcholine is not cell permeable and is rapidly degraded by acetylcholinesterase. Thus, endodermal cell types that might respond to acetylcholine are likely not exposed to acetylcholine by our protocol.

### Identification of *N. vectensis* nAChRs

We next set out to identify a comprehensive list of AChRs in *N. vectensis*. The initial draft of the *N. vectensis* genome predicted no mAChRs and 14 nAChRs, which likely arose by independent radiation [6, 9]. Recent single-cell RNA-sequencing studies in *N. vectensis* identified eight additional putative receptors (Table 1) [10]. The identification of new receptors suggested that the data set of nicotinic and muscarinic receptors in *Nematostella* was incomplete. We used BLAST searches to identify additional putative AChRs in the published genome and updated RNAseq data sets [9, 28]. We identified nine additional putative AChRs, bringing the total number of putative AChRs up to 31 (Table 1). Five of the thirty-one putative AChRs lacked the cysteine loop that is essential to form the acetylcholine-binding pocket [36, 49] and were, therefore, excluded from further investigation. Two were previously described in [6], two were identified in [10], and one we identified in our BLAST search (Table 1).

**Table 1:** Naming mechanisms for acetylcholine receptors

Our name	JGI: protein ID	NvERTx	[6] (Genome)	[10] (single-cell RNAseq.)	[27] (in situ)	JGI/ [28] (RNA-seq)
NvnAChRaA	31824	NvERTx.4.110720				31824/NvERTx.4.110720
NvnAChRaB	50745	NvERTx.4.143847				50745/NvERTx.4.143847
NvnAChRaC	11255	NvERTx.4.141630				11255/NvERTx.4.141630
NvnAChRaD	214990	NvERTx.4.137798	214990*	214990* (N/tlM)		
NvnAChRaE	91941	NvERTx.4.75581	91941*	91941* (N/tlM)		
NvnAChRaF	61041	NvERTx.4.55041				61041/NvERTx.4.55041*
NvnAChRaG	85724	NvERTx.4.110720		85724* (N/tlM)		
NvnAChRaH	85091	NvERTx.4.88235	85091*			
NvnAChRaI	205808	NvERTx.4.61521	205808*	205808* (N/tlM)		
NvnAChRaJ	199721	NvERTx.4.82387	199721*		ao145* (AO)	
NvnAChRaK	110265	NvERTx.4.147304	110265*		ao19* (AO)	
NvnAChRaL	205856	NvERTx.4.61144				205856/NvERTx.4.61144
NvnAChRaM	32916	NvERTx.4.161999				32916/NvERTx.4.161999
NvnAChRaN	205855	NvERTx.4.63946	205855			
NvnAChRaO	40806	NvERTx.4.31008				40806/NvERTx.4.31008
NvnAChRaP	87907	NvERTx.4.85000				87907/NvERTx.4.85000
NvnAChRaQ	198343	NvERTx.4.152487	198343*			
NvnAChRaR	198927	NvERTx.4.153628	198927*	198927* (N/tlM)		
NvnAChRaS	200917	NvERTx.4.111130	200917			
NvnAChRaT	40919	NvERTx.4.145232	40919			
NvnAChRaU	91696	NvERTx.4.52490	91696	91696* (N/M)		
NvnAChRaV	216224	NvERTx.4.141630		216224* (N/tlM/G)		
NvnAChRaW	57113	NvERTx.4.97064		57113 (N/G)		
NvnAChRaX	91371	NvERTx.4.56195		91371 (N/M)		
NvnAChRaY	79911	NvERTx.4.127752		79911 (N/M)		
NvnAChRaZ	97337	NvERTx.4.72427		97337* (N/M)		
Previously identified Ach. receptors that are removed in this study						
All missing cysteine loop	240779	NvERTx.4.60558	240779	240779 (tlM)		
	247410	NvERTx.4.76805	247410			
	22673	NvERTx.4.133515		22673 (N/tlM)		
	2281	NvERTx.4.56575		2281 (N/M)		
	205764	NvERTx.4.109818				205764/NvERTx.4.109818

A collection of previously identified, named, and characterized acetylcholine receptors gathered from the literature, BLAST searches of the JGI *Nematostella* genome (https://genome.jgi.doe.gov/Nemve1/Nemve1.home.html), and identified from BLAST searches of the NvERTx database (http://ircan.unice.fr/ER/ER_plotter/home). Confirmed nicotinic acetylcholine receptors are listed in the top portion of the table and were renamed *NvnAChRαA*-*Z*. Previously identified receptors that lack necessary features of nAChRs were excluded from analysis and are listed in the bottom portion of the table. Previously published expression patterns for acetylcholine receptors, and the publication they were described in, are indicated for each gene. *N* neurons, *M* muscle, *tlM* tentacular and/or longitudinal muscle, *G* gonad, *AO* apical organ. Asterisk indicates genes we obtained mRNA in situ hybridization patterns for in Fig. 4

**Fig. 2:**
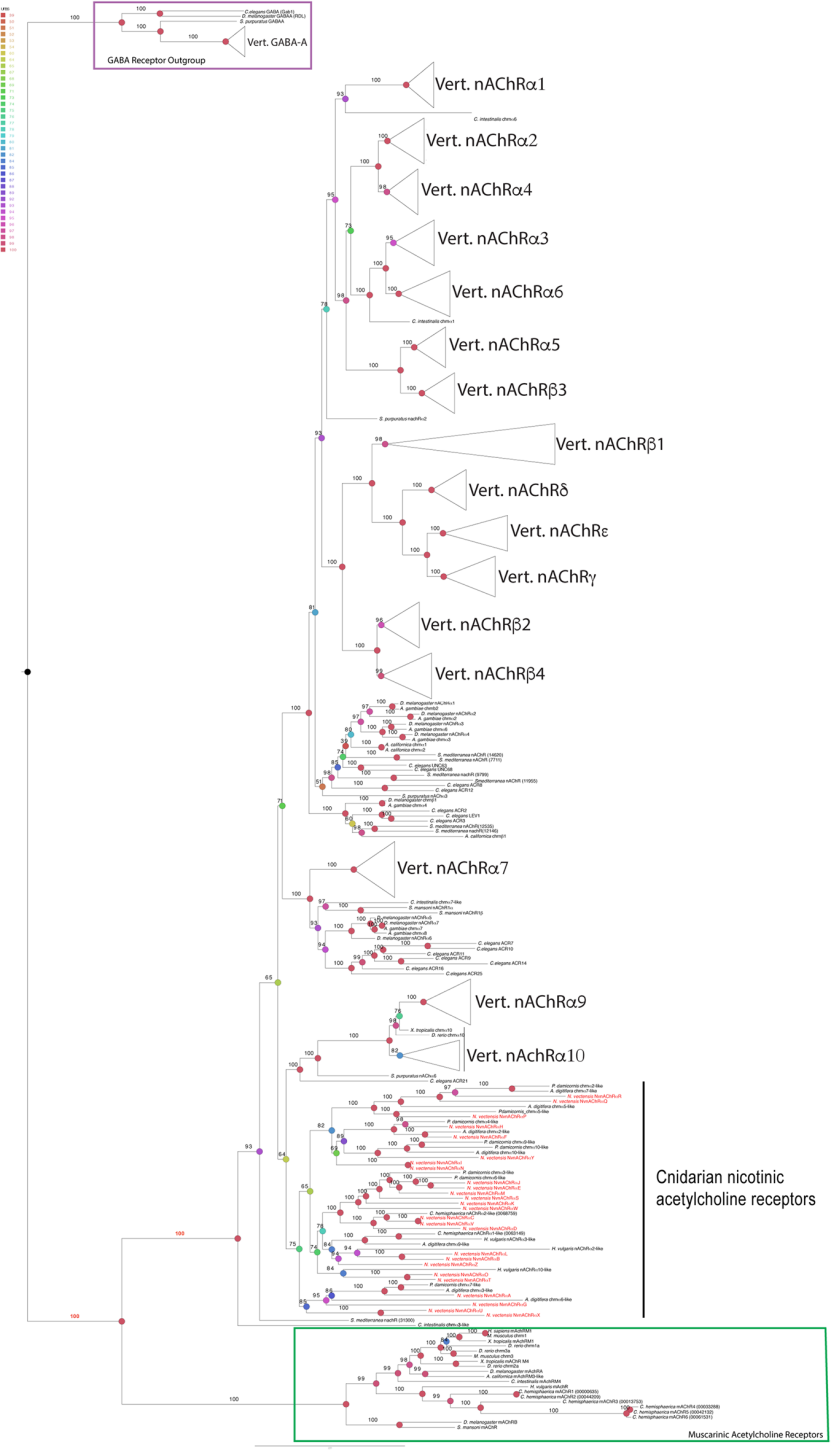
Phylogenetic analysis of unedited alignment of known and putative *AChR*s. Maximum likelihood phylogeny generated in IQ tree. The GABA receptors, indicated by a purple box, served as the out-group for our analysis. Muscarinic receptors are indicated by a green box. The ultrafast bootstrap values for critical nodes of interest are written in red. Tree generated using alignment in Additional file 9: Table S2. Heat map also indicates ultrafast bootstrap values. *N. vectensis* sequences are in red text on tips of tree branches

To determine if putative receptors were definitive AChRs, we performed Maximum Likelihood (IQ tree and RaxML) phylogenetic analyses using the remaining sequences and known mAChRs and nAChRs with ultrafast bootstrapping and rapid bootstrapping analysis, respectively. Following previous approaches, GABAergic receptors were used as the out-group to root the tree (Fig. 2, Additional files 1, 2, 3: Figures S1, S2, S3) [6, 34, 36]. Because there is little consensus in the literature about how to best edit the alignments for phylogenetic analysis, we used two previously published approaches. We used an unedited protein alignment (Additional file 9: Table S2) [34, 36], and an edit where we removed everything except the transmembrane domains (1–4) and the double cysteines necessary for acetylcholine binding (TD) (Additional file 10: Table S3) [35].

All *N. vectensis AChR*s clustered within known AChRs in trees generated using the unedited and TD alignments (UFBS = 100, 100) (BS = 100, 98) (Fig. 2, Additional files 1, 2, 3: Figures S1, S2, S3). None of the *N. vectensis* sequences clustered with the muscarinic receptors (Fig. 2; Additional files 1, 2, 3: Figures S1, S2, S3). In all analyses, the muscarinic receptors formed their own clade (UFBS = 100; BS = 100). The only cnidarian AChRs that clustered with the muscarinic genes were the known *Hydra* mAChR and six muscarinic genes identified in the *Clytia hemisphaerica* genome [26]. The lack of muscarinic receptors in *N. vectensis* was further supported by the fact that we did not identify the Q207(M3)/Q163(M2) and L204 motif necessary for G-coupled signaling in any of our *N. vectensis* sequences (Additional files 9, 10: Tables S2, S3) [50]. Interestingly, the phylogenetic placement of the muscarinic clade depended on the alignment edit and the phylogenetic analysis that was used. The IQtree and RaxML trees generated with the unedited alignment placed the muscarinic receptors as sister to the nicotinic receptors (Fig. 2 and Additional file 1: Figure S1, UFBS = 100, BS = 100), but phylogenies generated using the edited alignments placed the muscarinic clade as sister to the bilaterian nAchα3 clade (Additional file 2: Figure S2) or nested within the cnidarian receptors (Additional file 3: Figure S3). Since all analyses provided support that the *N. vectensis* genes are AChRs, and all analyses rule out that any of the *N. vectensis AChR*s are muscarinic, we conclude that all of the *N. vectensis AChR*s belong to the nAChR family.

We conclude that we have identified 14 previously undescribed nicotinic acetylcholine receptors in *N. vectensis* and that our data are consistent with previous reports suggesting independent radiation of cnidarian and bilaterian nAChRs. Three publications previously described *N. vectensis* cholinergic genes, but there is little consensus on the naming of these genes [6, 10, 27]. We have named the 26 genes *NvnAChRαA*–*NvnAChRαZ* (Table 1) so as not to confuse them with the established subunits that are classified using a number to define orthologous receptors. We also observed what appears to be some evidence of gene duplication within the *N. vectensis* nAChRs. We found several receptors, *NvnAChRαJ and NvnAChRαE, NvnAChRαC and NvnAChRαV*, and *NvnAChRαI* and *NvnAChRαN,* that are always sister with each other and could potentially be paralogs.

### nAChR activation is required for tentacular contraction

**Fig. 3:**
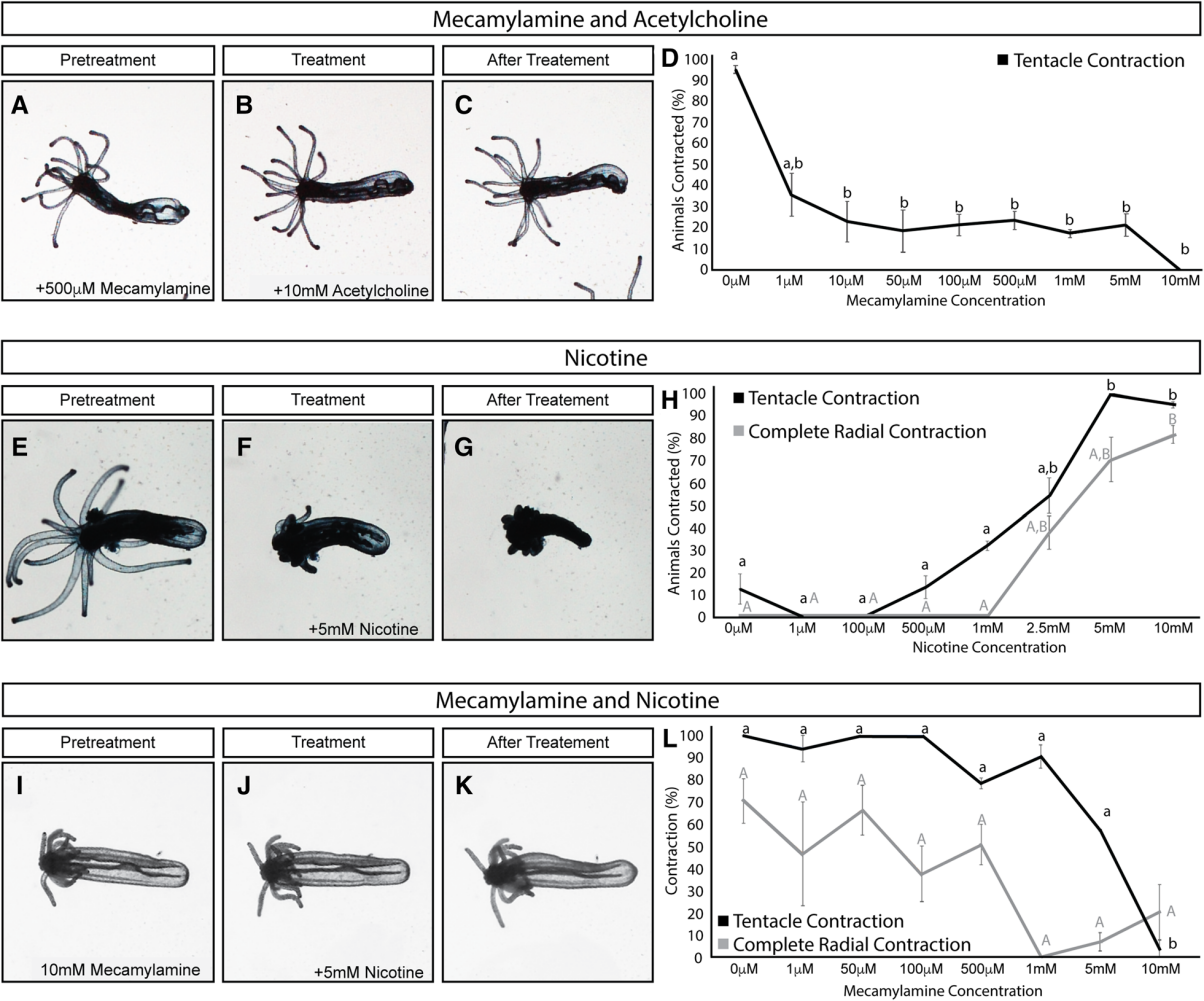
Pharmacological analysis of acetylcholine’s role in tentacular contractions. **A**–**D** Acetylcholine-induced tentacle contractions in 3-month-old polyps pretreated with 0 µM–10 mM mecamylamine. At 0 µM mecamylamine, 10 mM acetylcholine-induced tentacle contractions in 89 ± 2.32% of animals. This response dropped to 36 ± 10.13% of animals at 1 µM mecamylamine, though not significant (*p* = 0.095). Acetylcholine-induced tentacle contractions occurred in 20% of animals when pretreated with 10 µM–5 mM mecamylamine and dropped to 0 ± 0% in animals treated with 10 mM mecamylamine (p ≤ 0.05). **E**–**H** Nicotine-induced tentacle contractions occurred at 500 µM with 13.33 ± 5.09% and increased to 54 ± 7.99% at 2.5 mM, though these were not statistically different than the controls (*p* > 0.05). At 5 mM and 10 mM, nicotine-induced tentacle contractions occurred in 100 ± 0% and 98 ± 1.36% of animals, respectively, which was significantly different than the 0 µM–2.5 mM nicotine treatments (*p* ≤ 0.05). Nicotine-induced complete radial contractions were observed in 38 ± 7.43% of animals starting at 2.5 mM nicotine. At 5 mM and 10 mM nicotine, 70 ± 10.0% and 82 ± 4.05% of animals, respectively had complete radial contractions, these were significantly different than the controls (*p* ≤ 0.05). **I**–**L** Mecamylamine at lower concentrations (0 µM–1 mM) was not capable of blocking tentacular contractions. At 5 mM, nicotine-induced tentacle contractions were reduced to 57.22 ± 16.4%, though not significantly (*p* = 0.433). At 10 mM mecamylamine, nicotine-induced tentacle contractions were significantly reduced to 3.7 ± 6.8% (p ≤ 0.05). Body contractions were not significantly reduced throughout the mecamylamine gradient, though at high levels (1 mM–10 mM mecamylamine), we did see a trend towards a reduction in the number of body contractions. These data were calculated using a one-way ANOVA (**D**) Acetylcholine-induced tentacle contractions in 3-month-old polyps pretreated with 0 µM–10 mM mecamylamine *F*_*8,22*_ = 14.03, *p* < 0.001; (**H**, black) Tentacle contraction in the response to nicotine at different timepoints *F*_*7,17*_ = 43.15, *p* < 0.001. (**H**, gray) Body contraction in the response to nicotine at different timepoints *F*_*7,17*_ = 25.87, *p* < 0.001. (**L**, black) Tentacle contraction in 3-month-old polyps pretreated with 0 µM–10 mM mecamylamine *F*_*7,16*_ = 25.39, *p* < 0.001; (**l**, gray) Body contraction in 3-month-old polyps pretreated with 0 µM–10 mM mecamylamine *F*_*6,14*_ = 3.70, *p* < 0.05. Each experiment was performed *N* ≥ 3 times with an *n* ≥ 7/replicate. Points that do not share letters, either uppercase (in **H** and **L**) or lowercase (in **D**, **H**, **L**) are statistically different from each other

**Fig. 4:**
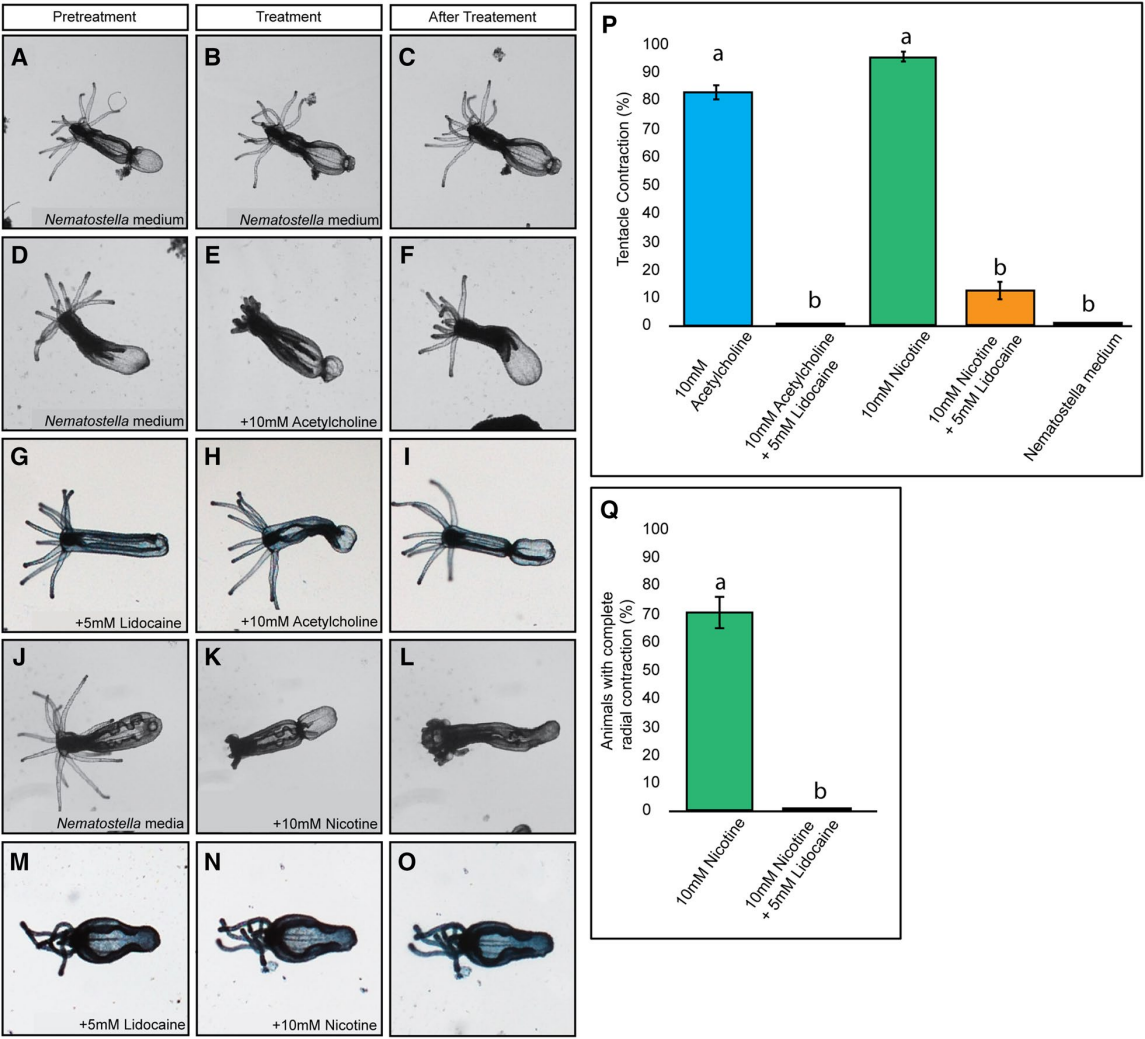
Lidocaine suppresses acetylcholine-mediated tentacular contractions. Pretreatment with 10 mM lidocaine resulted in loss of acetylcholine-induced tentacle contraction in all the juvenile polyps tested (**A**–**C**, **G**–**I**, **P**). This loss was statistically similar to controls (P–) (*p* ≥ 0.05). This was repeated in polyps pretreated with 10 mM lidocaine and then 10 mM nicotine (**M**–**O**, **P**). Samples pretreated with lidocaine had tentacular contractions that were significantly different from treatments with acetylcholine or nicotine alone (*p* ≤ 0.05%). Quantifications comparing the contraction of tentacles are summarized in **P**; bars that do not share letters indicate a significant difference between treatment groups. All images have the oral end directed to the left of the figure. *p* values were calculated using a student *t* test. Each experiment was performed *N* ≥ 3 times with an *n* ≥ 7/replicate

Because acetylcholine treatment can induce physiological responses in both ctenophores and poriferans that lack nAChRs and mAChRs [51, 52], we wanted to confirm that tentacular contractions are mediated by nAChRs. We used two approaches to show that nAChRs likely mediate tentacular contractions. First, we treated juvenile polyps with mecamylamine, an antagonist of nicotinic acetylcholine receptors that had been previously used in cnidarian animals [25]. Due to the delay in observed effects of lidocaine treatment [44], we chose to pre-block with mecamylamine for 20 min prior to the addition of acetylcholine. Although mecamylamine and other inhibitors have been used in cnidarians, there is a range of published concentrations with little explanation of how those values were obtained [20–23]. To address this we performed a dose response curve testing mecamylamine’s ability to block acetylcholine-induced tentacular contractions from 1 μM to 10 mM (Fig. 3A–D; Additional file 7: Video S3). In 36.5% of the animals, tentacular contractions occurred when treated with 1 μM mecamylamine (Fig. 3D). In 10 μM mecamylamine, only ~ 20% of the animals had tentacular contractions, which is similar to what was observed at all higher concentrations, and it is statistically different than the responses observed without mecamylamine treatment (Fig. 3A–D, *p* < 0.05). At mecamylamine concentrations of 1 mM and above, some animals involuted their oral opening resulting in a slight retraction of their tentacles into the mouth. However, their tentacles still remained elongated (Fig. 3I) and did not contract after the addition of acetylcholine (Fig. 3D; Additional file 11: Video S4). Based on the fact that neither acetylcholine nor nicotine (see below) showed any pharyngeal response, we predict that the involution is either an off-target stress response to high mecamylamine concentrations, or that acetylcholine activity is necessary to suppress pharyngeal involution. The second approach used to confirm that tentacular contractions are due to nAChR activity was to treat animals with nicotine, which is the namesake agonist of nAChRs (rev. [53]. We performed a dose response similar to that conducted with acetylcholine (Fig. 3H). We observed the first hints of activation with 1 mM nicotine, though it was not significantly different than the NGM control (*p* = 0.409). By 5 mM, nearly 100% of animals responded by contracting their tentacles (Figs. 3E–H, 4J–L, P, Additional file 12: Video S5, *p* < 0.05). This is consistent with reports in other systems that described nicotine as having a higher affinity for some nAChRs than acetylcholine [54]. Animals treated with nicotine did not relax their tentacles after contraction, presumably due to the inability of acetylcholinesterase to metabolize nicotine [55] (Fig. 3F, G). Surprisingly, nicotine also induced strong radial contractions (Fig. 3G), which are mediated by radial muscles in the endoderm. We observed a complete contraction of the radial muscles along the entire length of the oral–aboral axis in 71% and 81% of animals ~ 4 min after the addition of 5 or 10 mM nicotine, respectively (Fig. 3G). Further observations up to 20 min did not reveal any additional contractions (data not shown). At least two possibilities explain the differences between acetylcholine and nicotine treatments. First, nicotine is cell permeable, but acetylcholine is not. It is possible that there are acetylcholine responsive muscles in the endoderm that are being acted on by nicotine that is diffusing into the endodermal layer. Acetylcholine on the other hand is both unable to diffuse into the endoderm, and it is actively degraded by acetylcholinesterases, which are known to be widely expressed [10, 47]. The second possibility is that the secondary contractions are due to some off-target effect by nicotine. To address these possibilities, we replicated our mecamylamine dose response with 5 mM nicotine. We found that 10 mM mecamylamine was able to block tentacular contractions, and reduced complete radial contractions, though not significantly (*p* = 0.400) (Fig. 3I–L). To further confirm the potential role of nAChRs in regulating body contraction, we quantified the percentage of animals that performed peristaltic initiation or peristaltic wave contractions in different mecamylamine concentrations (Additional file 8: Figure S4B). Peristaltic initiation is a localized radial contraction that looks like a pinching-in around the entire animal. The peristaltic wave is a wave of radial contractions that propagates aborally away from the peristaltic initiation contraction. Untreated animals have at least one peristaltic wave/minute (Additional file 8: Figure S4A). Concentrations of mecamylamine above 500 μM result in a significant reduction of peristaltic waves (*p* ≤ 0.05), and the radial peristaltic initiation contractions are trending towards being reduced. However, the reduction in peristaltic initiation contractions was not statistically significant (*p* ≥ 0.05) (Additional file 8: Figure S4B). The ability of mecamylamine to block tentacular contractions induced by nicotine supports the hypothesis that tentacular contractions are mediated by activating nAChRs. Furthermore, the ability of mecamylamine to block the complete radial contractions induced by nicotine, suppress normal peristaltic initiation contractions, and block peristaltic wave contractions suggests that nAChRs also contribute to regulating radial muscle contractions in *N. vectensis.* In no instances did acetylcholine or nicotine induce contraction of the oral–aboral axis, arguing that nAChRs do not mediate oral–aboral axis contraction in *N. vectensis*.

#### Lidocaine treatment suggests that acetylcholine-induced muscle contractions are due to neuronal activation

In bilaterians, acetylcholine functions at chemical synapses in neuronal and neuromuscular synapses. Although acetylcholine has been shown to regulate contractions of cnidarian musculature, it is not clear if acetylcholine activates neurons, muscles, or both [20–23, 56, 57]. To gain insight about what cell types were being activated by acetylcholine, we treated animals with lidocaine, a known inhibitor of voltage-gated sodium channels that has been shown to effectively block *N. vectensis* voltage-gated sodium channels [44, 58]. Voltage-gated sodium channels are critical for neuronal excitation and are not expressed in *Nematostella* musculature [10, 44]. We, therefore, reasoned that if nAChRs are activated in myoepithelial cells, treatment with lidocaine should have no effect on tentacle contractions following acetylcholine application. However, if nAChRs are activated in neuronal cells that promote contractions of tentacular musculature, then we predicted that treatment with lidocaine should suppress tentacular contractions. Previous work has shown that 1–10 mM is capable of blocking sodium channels expressed in heterologous systems, and that pretreatment of *N. vectensis* polyps with 5 mM lidocaine is sufficient to paralyze animals [44]. Pretreatment with 5 mM lidocaine resulted in a loss of acetylcholine-induced tentacle contractions. No animals contracted in the presence of lidocaine (Fig. 4D–I, P) (Additional file 14: Video S6) compared to the 83% of animals that contracted in the acetylcholine treated control group. This loss of tentacular contraction was replicated in nicotine treated animals as well (Fig. 4M–O, P) Additional file 15: Video S7. Lidocaine also blocked radial contractions induced by nicotine (Fig. 4M–O, Q) Additional file 15: Video S7. The ability of lidocaine to block both acetylcholine and nicotine-induced tentacle contractions suggest that acetylcholine mediates contractions by activating nAChRs in neurons rather than myoepithelial cells. However, because we cannot rule out that lidocaine does not have an off-target effect in *N. vectensis* musculature, patch clamp or other single-cell resolution studies should be done to confirm that acetylcholine does not directly activate tentacular musculature. Regardless of exactly what cell types are activated, our findings and previous observations strongly argue nAChR function in neuronal/neuromuscular communication is widespread in cnidarians, which implies that it is a feature of the ancestral cnidarian nAChR. Because both cnidarian and bilaterian nAChRs have roles in neuronal/neuromuscular communication, it is likely that a neuronal role for nAChRs emerged at or near the base of their evolution.

#### Determining spatiotemporal expression of acetylcholine receptors

**Fig. 5:**
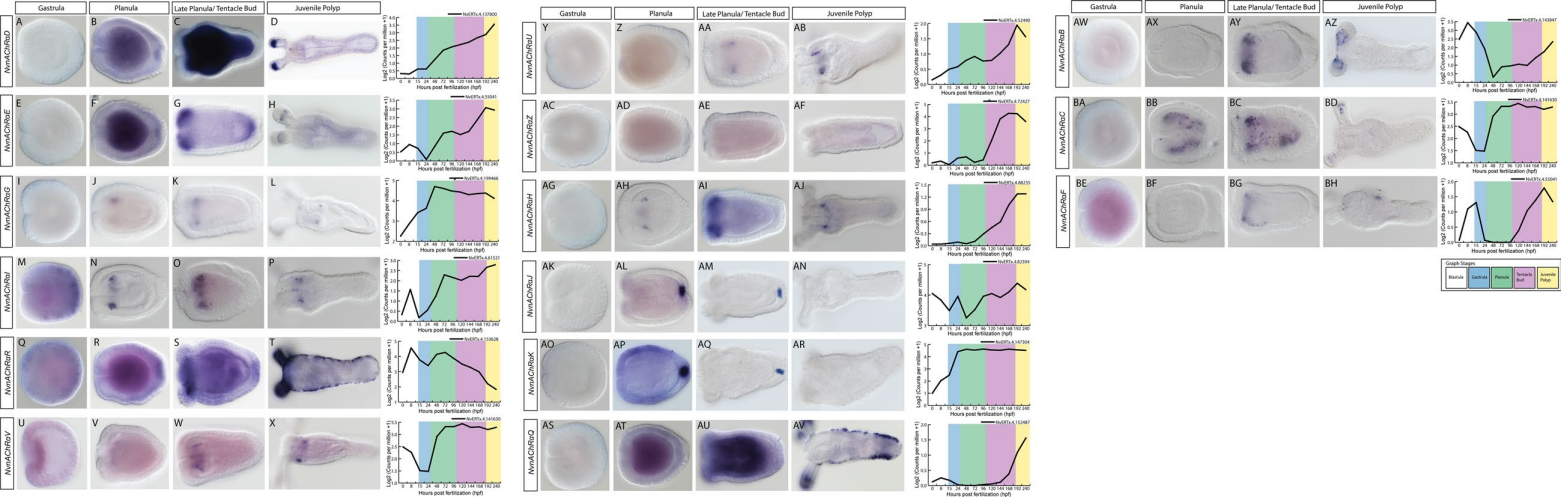
Expression patterns of *N. vectensis* nicotinic acetylcholine receptor genes. *NvnAChRαD* was broadly expressed in the planula and tentacle bud stages, but then became restricted to the endodermal tips of the tentacles (**A**–**D**). *NvnAChRαE* expression begun during the planula stages and then became restricted to the tentacle buds at the tentacle bud stage. By the polyp stage, expression was restricted to a band in the tentacles (**E**–**H**). *NvnAChRαG* expression begun in the planula within the developing pharynx, and this pharyngeal expression was maintained into the juvenile polyp stage (**I**–**L**). *NvnAChRαI* expression started in a scattered salt and pepper pattern at the aboral end, then restricted to the developing pharynx in the planula and was maintained here in the juvenile polyp (**M**–**P**). *NvnAChRαR* expression started broadly within the gastrula, planula, and tentacle bud stages, and this broad expression was maintained in the endoderm of the juvenile polyps (**Q**–**T**). *NvnAChRαV* expression started at the late planula stage within the pharynx, and expression was maintained there in the juvenile polyps (**U**–**X**). *NvnAChRαU* expression started at the late planula/early tentacle bud stage in the developing pharynx, and was maintained there in the juvenile polyps (**Y**–**AB**). We did not observe any expression of *NvnAChRαZ,* despite RNA-sequencing data suggesting expression after 144hpf (**AC**–**AF**). Expression of *NvnAChRαH* started at the planula stage in the developing pharynx, and was maintained there in the juvenile polyps (**AG**–**AJ**). *NvnAChRαJ* and *NvnAChRαK* expression both started in the planula stages, continued being expressed into the tentacle bud stage at the apical tuft, and was lost by the juvenile polyp stage (**AK**–**AR**). *NvnAChRαQ* was first broadly expressed at the planula and tentacle bud stages, then became restricted to endodermal tentacle and aboral ectoderm (**AS**–**AV**). A scattered salt and pepper expression of *NvnAChRαB* was observed in the developing tentacles, and the tentacles of the juvenile polyps (**AW**–**AZ**). *NvnAChRαC* expression was observed at the planula stages as a scattered salt and pepper pattern in the endoderm until the juvenile polyp stage, when expression was restricted to the tentacle tips (**BA**–**BD**). *NvnAChRαF* was expressed at the late planula stages within a few cells of the developing tentacles; this expression became restricted to a cluster of cells at the basal end of the tentacles (**BE**–**BH**). Graphs at the end of each row consist of the temporal expression according to previously published RNA-seq. data [28]. Within the graphs, white = blastula, blue = gastrula, green = planula, red = late planula/tentacle bud, and yellow = juvenile polyp stages. All images have the oral end directed to the left

To further investigate the potential role of nAChRs, we attempted to better describe the spatial expression patterns of each receptor. In particular, we were interested in determining if expression patterns of nAChRs would provide support for both neuronal and non-neuronal functions in cnidarians. Single-cell sequencing identified six nAChRs as being expressed in neurons, tentacles, or longitudinal muscles, but did not resolve which muscle type specifically [10]. Because these muscle are located in different regions and derived from different tissue layers during development, we attempted to resolve which muscle type the nAChRs were expressed in using mRNA in situ hybridization. We were able to clone all of nAChRs predicted to be in the tentacular and longitudinal musculature (*NvnAChRαD*, *NvnAChRαE*, *NvnAChRαG*, *NvnAChRαI*, *NvnAChRαR*, and *NvnAChRαV*). *NvnAChRαD*,*E*, and *R* were all expressed in the tentacles (Fig. 5D, H, T). *NvnAChRαD* and *NvnAChRαE* are both expressed in the ectoderm near the distal end of the tentacles (Fig. 5D, Additional file 13: Figure S5). During larval stages, *NvnAChRαD* and *NvnAChRαE* are expressed in the developing endoderm (Fig. 5B, C, F, and G), and *NvnAChRαE* is expressed in the developing tentacle buds and is enriched in the endoderm at the aboral pole (Fig. 5G). *NvnAChRαR* is expressed throughout the tentacle ectoderm (Fig. 5T). The broad pattern of *NvnAChRαR* throughout the tentacle ectoderm indicates that it is likely expressed in muscles that control tentacle contraction, but it also suggests that expression is not specific to the muscles. *NvnAChRαR* also appears to have low-level ubiquitous expression during larval and tentacle bud stages (Fig. 5R, S). The broad expression patterns for *NvnAChRαD*, *E*, and *R* are consistent with each gene having roles in cell signaling in addition to any putative neuronal roles. During development and in juvenile polyps, *NvnAChRαI*, *NvnAChRαG*, and *NvnAChRαV* are expressed in cells around the pharynx (Fig. 5L, P, X). *NvnAChRαV* expression is reported in the developing gonad [10], which is consistent with the observed mRNA in situ hybridization pattern (Fig. 5X). Because *NvnAChRαI* and *NvnAChRαG* were not reported in the gonads, it is not clear if they represent false negatives in [10] or if they are expressed in a distinct population of cells. It should be noted that the expression patterns of *NvnAChRαV*, *NvnAChRαI*, and *NvnAChRαG* are not identical. *NvnAChRαI* and *NvnAChRαG* are expressed in smaller domains than that of *NvnAChRαV* (Compare Fig. 5L, P, X). We also identified the expression patterns of two genes which were broadly reported to be expressed in muscle cells, *NvnAChRαU* and *NvnAChRαZ* [10]. The *NvnAChRαU* mRNA in situ pattern is similar to *NvnAChRαG*, *NvnAChRαI*, and *NvnAChRαV* within the developing and juvenile polyp pharynx (Fig. 5AA, AB). We observed low levels of ubiquitous expression for *NvnAChRαZ* in planula stages (Fig. 5AD), but were unable to detect expression in the polyp despite it being reported to be expressed at high levels after 114hpf (Fig. 5AF) [28]. The lack of obvious expression in both neurons and muscle for *NvnAChRαG*, *NvnAChRαI*, *NvnAChRαV*, and *NvnAChRαU* at any stage of development was surprising. One possible explanation for the discrepancy is that these genes may be expressed at low levels in neurons and/or muscle making detection by mRNA in situ hybridization difficult in those cell types. The mRNA in situ patterns we did obtain suggests that, although expression in musculature and neurons are described for *NvnAChRαD*, *NvnAChRαE*, *NvnAChRαR*, and *NvnAChRαZ,* these receptors are likely not exclusively expressed in neurons or muscle cells.

We characterized the expression of receptors not known to be expressed in neurons or muscle (*NvnAChRαH*, *NvnAChRαJ*, *NvnAChRαK*, *NvnAChRαQ*, *NvnAChRαB*, *NvnAChRαC.* and *NvnAChRαF).* Interestingly, *NvnAChRαH* was expressed in a subset of pharyngeal cells, which was similar to the expression observed for *NvnAChRαG*, *NvnAChRαI*, *NvnAChRαU*, and *NvnAChRαV* (Fig. 5AG–AJ). *NvnAChRαH* was also expressed broadly in the endoderm of forming tentacle buds, which is consistent with a role in cell signaling or patterning within those cell types (Fig. 5AI). *NvnAChRαB* is expressed in a scattered salt and pepper pattern in developing and formed tentacles (Fig. 5AW–AZ). *NvnAChRαC* is first expressed in a scattered salt and pepper pattern within the planula endoderm (Fig. 5BB). At the tentacle bud stage, expression became restricted to the endodermal tissue beneath the tentacle tips (Fig. 5BD). *NvnAChRαF* expression started at the late planula/tentacle bud stage in the endodermal tissue, where the tentacles had begun to develop (Fig. 5BG). At the juvenile polyp stage, *NvnAChRαF* expression became restricted to a few cells near the base of the tentacles (Fig. 5BH). The scattered salt and pepper pattern of *NvnAChRαB*, *C*, and *F* likely represents expression in differentiated cell types, but none of these genes is reported to be expressed in neurons [10]. We also found that *NvnAChRαQ*, which was not identified in previous single-cell RNA sequencing, was expressed in the tentacles and in the body column, sharing some overlap with the ectodermal expression of *NvnAChRαR*, *D*, and *E* (Fig. 5A–H, AS–AV). *NvnAChRαJ* and *NvnAChRαK* (aka, 199721/ao145 and 110265/ao19, respectively) were expressed in the apical organ ectoderm of planula larvae, which is consistent with their previously described expression (Fig. 5AK–AR) [27]. The apical expression was subsequently lost in juvenile polyps (Fig. 5P, T). Acetylcholine signaling has been linked to metamorphosis in free-swimming bivalve larvae, and the apical organ is linked to settlement and metamorphosis in some corals, suggesting that the apical organ expression may indicate a conserved role for acetylcholine in anthozoan cnidarian metamorphosis [59, 60].

Seven of the fourteen expression patterns we recovered (*NvnAChRαD*, *E*, *R*, *H*, *J*, *K*, *Q*, and *V*) had expression patterns that are more consistent with cell signaling or developmental patterning than restricted roles in neurons and/or muscle. Bilaterian AChRs function within multiple non-neuronal cell types, neuronal cells, and muscles cells. Although we were unable to subclone and generate probes for all of the predicted *NvnAChRs*, our expression data argue that both neuronal and non-neuronal functions for nicotinic acetylcholine receptors in bilaterians were likely present in the cnidarian–bilaterian ancestor.

## Discussion

### nAChRs present in the cnidarian–bilaterian ancestor likely underwent lineage-specific radiations

Our phylogenetic analyses strongly suggest that *N. vectensis* has 26 nAChRs, and that they are more closely related to other cnidarian nAChRs than they are to bilaterian nAChRs (Fig. 2; Additional files 1, 2, 3: Figures S1, S2, S3). This supports the previous hypothesis that nAChRs radiated independently in cnidarian and bilaterian lineages. Independent radiation is also likely when considering that nAChRs first arose within, or just prior to, the emergence of the cnidarian–bilaterian common ancestor [6]. We were unable to detect any nAChRs in BLAST searches of published pre-cnidarian/bilaterian genomes, and no nAChRs have been described outside of cnidarians or bilaterians. Additional evidence for independent radiation is that the protostome–deuterostome nAChRs largely form independent clades, which supports the hypothesis that a small number of nAChRs were present in the protostome–deuterostome common ancestor [34, 35]. A similar phenomenon has been suggested in muscarinic family evolution, which suggests independent expansion of muscarinic receptors in protostome and deuterostome lineages [61]. Compared to other cnidarian species, it appears that a number of gene duplication events have occurred throughout *N. vectensis* evolution, generating multiple paralogs not observed in the other lineages (Fig. 2; Additional files 1, 2, 3: Figures S1, S2, S3).

Previous work identified only one cnidarian muscarinic receptor in *Hydra* [18]. We found six muscarinic acetylcholine receptors in the *Clytia hemisphaerica* genome (Fig. 2, Additional files 1, 2, 3: Figures S1, S2, S3) [26]. Interestingly, no cnidarian muscarinic acetylcholine receptors have been identified outside of the hydrozoans. This suggests that muscarinic receptors were present in the cnidarian–bilaterian ancestor, but lost in the anthozoans. However, an improved understanding of cnidarian muscarinic receptors is required to better determine the gain–loss patterns and evolution of mAChRs in animals.

### Neuromodulator activity likely evolved coincidently with emergence of nAChRs

Acetylcholine is a neuromodulator at chemical synapses in bilaterians, and this neuromodulatory activity occurs through both nicotinic and muscarinic receptors. We and others have shown that acetylcholine, and other acetylcholine receptor agonists, elicit contractions in multiple cnidarian species (Figs. 1, 3A–D, 4D–F, P) [22, 23, 24]. More specifically, recent work has shown that acetylcholine acts on the cnidocyte synapses to modulate cnidocyte firing [25]. We showed that the nAChR antagonist mecamylamine suppresses tentacular contractions in the presence of acetylcholine, and that nicotine, an agonist of nAChRs, induces tentacle contractions (Fig. 3). Mecamylamine also suppressed peristaltic wave contractions (Additional file 8: Figure S4B) and nicotine mediated radial contractions (Fig. 3G–H). Finally, treatment with lidocaine, the voltage-gated sodium channel antagonist, also suppressed all contractions in the presence of acetylcholine or nicotine (Fig. 4). Together these data suggest that nAChRs function in neuronal and/or neuromuscular communication to regulate muscle contractions in cnidarians. The widespread role for acetylcholine-mediated contractions in cnidarians (Figs. 1, 3, 4) [20–23], its conserved expression in neurons and muscles (Fig. 5) [10, 11], and the fact that 11 of the *N. vectensis* receptors are expressed in neurons and/or muscle [10], make for a strong argument that the neuronal role of nAChRs was present in stem cnidarians. The presence of widespread neuronal function for nAChRs in both cnidarians and bilaterians suggests that neuronal functions of nAChRs arose coincidently with their emergence at or near the base of nAChR evolution prior to the cnidarian–bilaterian divergence.

The inability of nicotine or acetylcholine to induce contractions in the presence of lidocaine suggests that tentacular contractions, and radial contractions associated with peristaltic waves, are mediated by nAChR activity in neurons rather than musculature. Lidocaine inhibits the voltage-gated sodium channels necessary for action potentials, and has been shown to block the activity of *N. vectensis* channels [44]. In the *N. vectensis* single-cell RNAseq data set, no voltage-gated sodium channels were identified in muscle cells [10]. One caveat is that lidocaine has been shown to impact smooth muscle contractions [62] in bilaterians. However, the smooth muscle contractions are inhibited by lidocaine acting on muscarinic AChRs [63]. Lidocaine does not disrupt nAChRs, there are no muscarinic receptors in the *N. vectensis* genome, and the two *N. vectensis* genes that cluster most closely with muscarinic receptors in a subset of our phylogenies (*NvnAChRαA* and *NvnAChRαG*) are not expressed in tentacles or radial muscle cells (Table 1; Fig. 5I–L) [10]. Together, these observations allow us to argue that the inhibition of contractions is not due to inhibition of tentacle musculature by lidocaine. However, we acknowledge that a yet unidentified protein expressed in muscles could be inhibited by an off-target of lidocaine, or that lidocaine could inhibit *N. vectensis* NvnAChRs. To date, evidence arguing for direct activation of muscle by acetylcholine does not exist. As such, experiments aimed at characterizing nAChR function at a cellular resolution are necessary to map which cell type(s) requires acetylcholine activation to promote muscle contractions in *N. vectensis*.

We had hoped that a better characterization of nAChR expression would help to determine if nAChR activity in neurons or muscles induced contractions in *N. vectensis.* We observed expression of *NvnAChRαD, NvnAChRαE,* and *NvnAChRαR* within the tentacle ectoderm, where the muscles that control contraction reside (Fig. 5A–H, Q–T, Additional file 13: Figure S5) [10, 48]. *NvnAChRαD* and *NvnAChRαE* both had limited overlap with the tentacular longitudinal muscles, and *NvnAChRαR* was expressed throughout the tentacles in a broad domain that has both neuron and muscle cells. None of the *NvnAChR* genes were expressed in a pattern consistent with being in radial muscle, although, based on single-cell RNAseq data, *NvnAChRαX*–*Z* and *NvnAChRαU* are both candidates to be expressed in radial muscle [10]. *NvnAChRαC* was expressed in a pattern consistent with potentially being in endodermal neurons that might regulate radial muscles, but *NvnAChRαC* was not identified in neurons by single–cell RNAseq [10]. One concern is that mRNA in situ hybridization data may not detect low-level expression in neurons or muscle. If nAChRs have a slow turnover at the synapse, high levels of mRNA would not be required to maintain enough receptors. Thus, more detailed analysis of single-cell RNAseq data coupled with functional studies at single-cell resolution (see above) will be necessary to determine which cells require acetylcholine to induce contractions of *N. vectensis* musculature. Nonetheless, our data in total, coupled with evidence from a range of cnidarian species, point to a conserved role for nAChRs in neuronal communication.

#### Non-neuronal roles of acetylcholine within *N. vectensis*

Acetylcholine signaling between cells predates the origin of nicotinic and muscarinic AChRs. The enzymes necessary to synthesize acetylcholine are widespread in both the metazoans, plants, and single-celled organisms [16, 64, 65], and it has been purified from bacteria, fungi, plant, and metazoan extracts [64, 66]. Clear responses to acetylcholine have been documented in metazoans, fungi, and plants [5, 67–70]. In the fungi, *Candida albicans*, acetylcholine inhibits biofilm formation [69]. Within plants, acetylcholine regulates growth, cellular differentiation, water homeostasis, and photosynthesis [67, 68]. In ctenophores and poriferans, treatment with acetylcholine increased luminescence excitation and prolonged cycles of rhythmic contraction, respectively [51, 52]. These observations make for a strong argument that the non-neuronal acetylcholine signaling predated the neuronal functions described for nAChRs and mAChRs. However, it is unclear if the non-neuronal functions for bilaterian nAChRs and mAChRs arose within the bilaterians or nearer the base of nicotinic and muscarinic receptor evolution.

To gain a better understanding about potential non-neuronal functions of nAChRs, we characterized spatiotemporal expression patterns of 16 of the 26 *N. vectensis* nAChRs. We found that seven of the genes we were able to clone (*NvnAChRαD*, *E*, *R*, *H*, *J*, *K*, *Q*, and *V*) (Fig. 5) had patterns that are consistent with non-neural functions. Many of these genes were expressed in dynamic developmental patterns and were broadly expressed in a tissue during larval stages (such as in the tentacle bud, the apical organ, or pan-endodermally), but then expression subsided. The broad non-cell-type-specific pattern that is dynamic during development is more consistent with a role in patterning than it is with a defined neuronal or muscle function. However, it does not eliminate the notion that those genes could be functioning at chemical synapses in the neurons and/or muscles in which they are expressed. *NvnAChRαB, NvnAChRαC,* and *NvnAChRαF,* all had a scattered salt and pepper expression in the juvenile polyp pharynx (Fig. 5AW–BH). None of these genes were identified as neuronal, muscular, cnidocyte, or gland cells in the single-cell RNA-sequencing data set. It is unclear if cells expressing *NvnAChRαB, NvnAChRαC,* and *NvnAChRαF* represent an unknown cell type or represent false negatives in the single-cell RNA-sequencing data. Two receptors have expression consistent with a role for acetylcholine in metamorphosis. *NvnAChRαJ* and *NvnAChRαK* are expressed in the apical organ in late stage planula (Fig. 4O, S) [27]. Acetylcholine has been found to be involved in the settlement and metamorphosis of free-swimming bivalve larvae including *Pinctada maxima*, *Crassostrea gigas*, *Mytilus edulis*, and *Mytilus galloprovincialis* as well as within coral planula larvae [59, 60]. The presence of broad expression patterns that are consistent with roles in development for 7 of the 15 genes tested here suggests that non-neuronal functions likely existed for the ancestral nAChRs that radiated in the cnidarian and bilaterian lineages.

## Conclusions

Our data, coupled with the previous observations, indicate that nicotinic acetylcholine receptors function in non-neuronal cell signaling and neuromodulation in *N. vectensis.* Because nAChRs evolved in the cnidarian–bilaterian ancestor, our work suggests that both the non-neuronal functions and neuromodulatory roles of nAChRs emerged coincidently with, or closely after, ancestral nAChR(s) evolved. Further work must be done to definitively characterize, where and what cell types NvnAChRs are activating to induce muscle contractions and to identify non-neuronal NvnAChR functions.

## Supplementary information

**Additional file 1: Figure S1.** Phylogeny generated in RaxML using an unedited alignment. RaxML generated maximum likelihood phylogeny determining the relationship of potential cnidarian acetylcholine receptors to the known bilaterian nicotinic and muscarinic acetylcholine receptors. The GABA receptors, indicated by a purple box, served as the out-group for our analysis. Muscarinic receptors are indicated by a green box. The bootstrap values for critical nodes of interest are written in red. Tree generated using alignment in Additional file 9: Table S2. Heat map also indicates bootstrap values. *N. vectensis* sequences are in red text on tips of tree branches.

**Additional file 2: Figure S2.** Phylogeny generated in IQ tree using an edited alignment that includes the transmembrane domains and double cysteines. IQ-tree generated maximum likelihood phylogeny determining the relationship of potential cnidarian acetylcholine receptors to the known bilaterian nicotinic and muscarinic acetylcholine receptors. The GABA receptors, indicated by a purple box, served as the out-group for our analysis. Muscarinic receptors are indicated by a green box. The ultrafast bootstrap values for critical nodes of interest are written in red. Tree generated using alignment in Additional file 10: Table S3. Heat map also indicates ultrafast bootstrap values. *N. vectensis* sequences are in red text on tips of tree branches.

**Additional file 3: Figure S3.** Phylogeny generated in RaxML using an edited alignment that includes the transmembrane domains and double cysteines. RaxML generated maximum likelihood phylogeny determining the relationship of potential cnidarian acetylcholine receptors to the known bilaterian nicotinic and muscarinic acetylcholine receptors. The GABA receptors, indicated by a purple box, served as the out-group for our analysis. Muscarinic receptors are indicated by a green box. The bootstrap values for critical nodes of interest are written in red. Tree generated using alignment in Additional file 10: Table S3. Heat map also indicates bootstrap values. *N. vectensis* sequences are in red text on tips of tree branches.

**Additional file 4: Table S1.** Primers used for amplification. Each gene is listed as named in the paper and with the *N. vectensis* JGI gene ID name. Forward primer is on top and reverse primer is on the bottom for each gene listed. All primers are 5′ → 3′.

**Additional file 5: Video S1.** Treatment with *N. vectensis* medium. Animals were treated with *N. vectensis* growth medium when indicated in the video. Red arrows highlight examples of animals that had no tentacular contractions. Magenta arrow highlights example of animal that contracted. The video is 1× magnification and 7 frames per second.

**Additional file 6: Video S2.** 10 mM acetylcholine induces tentacle contractions. Acetylcholine was added when indicated in the video and induced immediate tentacular contractions. 10× Magnification is used to highlight that tentacles contracting along proximal distal axis. The video is 7 frames per second.

**Additional file 7: Video S3.** Treatment with 10 mM acetylcholine. Treatment with acetylcholine, added when indicated in the video, induced tentacle contractions in *N. vectensis*. Red arrows highlight examples of animals that had tentacular contractions. Magenta arrow indicates an example of animal that partially contracted. Video was taken at 1× magnification at 4 frames per second.

**Additional file 8: Figure S4.** Quantifications of radial contractions and peristaltic waves in the presence of *N. vectensis* medium, acetylcholine, nicotine, and mecamylamine. (A) Treatment with acetylcholine did not induce a statistically significant difference in the number of animals with peristaltic initiation contractions (black) or peristaltic wave contractions (gray) (p ≥ 0.05). Treatment with nicotine alone resulted in complete radial contractions, thus no peristaltic wave contractions or peristaltic initiation contractions occurred. (B) Treatment with mecamylamine at 1 mM reduced the percentage of animals with peristaltic initiation contractions from ~ 91.53 ± 4.33% to 61.1 ± 16.6%. The peristaltic initiation contractions continued to drop to 42.6 ± 8.24% with 10 mM mecamylamine. A similar drop was observed in the percentage of animals who performed peristaltic wave contractions from 91.53 ± 4.33% at 500 µM to 25.39 ± 5.7% at 1 mM mecamylamine. The radial contractions were reduced to 0 ± 0% when treated with 10 mM mecamylamine. Each experiment was performed N > 3 times with an n ≥ 7/replicate. *p* values were calculated using a student *t* test for (A) and a one-way ANOVA for (B). (B, black) Peristaltic initiation contractions in 3-month-old polyps pretreated with 0 µM–10 mM mecamylamine *F*_*7,16*_ = 7.06, p < 0.005 (B, gray) Peristaltic wave contractions in 3-month-old polyps pretreated with 0 µM–10 mM mecamylamine *F*_*8,18*_ = 60.56, p < 0.001. Points that do not share letters, either uppercase or lowercase (B), are statistically different from each other.

**Additional file 9: Table S2.** Unedited amino acid alignment. The unedited protein alignment of confirmed bilaterian muscarinic and nicotinic acetylcholine receptors, GABAergic receptors, a confirmed *Hydra* muscarinic acetylcholine receptor, and the potential *N. vectensis* nicotinic acetylcholine receptors and cnidarian nicotinic acetylcholine receptors. Asterisks indicate the cysteines that make up the cysteine loop that defines the family.

**Additional file 10: Table S3.** Edited amino acid alignment consisting of transmembrane domains and double cysteines necessary for acetylcholine binding. A protein alignment of confirmed bilaterian muscarinic and nicotinic acetylcholine receptors, GABAergic receptors, a confirmed *Hydra* muscarinic acetylcholine receptor, a potential *N. vectensis* nicotinic acetylcholine receptor and a cnidarian nicotinic acetylcholine receptor. Protein sequences were edited to consist of the four conserved transmembrane domains (TM1–TM4) and the double cysteines necessary for acetylcholine binding (denoted by asterisks).

**Additional file 11: Video S4.** Pretreatment with 10 mM Mecamylamine inhibits tentacle contractions normally induced by 10 mM acetylcholine. Acetylcholine was added when indicated. The pretreatment with mecamylamine inhibited tentacular contractions. Magenta arrow indicates an animal that had radial contractions, but no peristaltic wave, when pretreated with 10 mM mecamylamine. Red arrows highlight examples of animals without tentacular contractions. Video was taken at 1× magnification at 4 frames per second.

**Additional file 12: Video S5.** Treatment with 10 mM Nicotine. Nicotine was added when indicated in the video. Red arrows highlight examples of animals that had tentacular contractions. Red arrows highlight examples of animals that had tentacular contractions and complete radial contractions. Magenta arrow highlights an animal that had partial tentacle contractions and no complete radial contraction. The video was taken at 1× magnification and 7 frames per second.

**Additional file 13: Figure S5.** Expression of *NvnAChRαD* and *NvnAChRαE* in the tentacles. Expression of *NvnAChRαD* and *NvnAChRαE* are restricted to the tentacular ectoderm. Dotted line separates the ectoderm and endodermal tissue layers. ec = ectoderm, en = endoderm.

**Additional file 14: Video S6.** Pretreatment with 10 mM Lidocaine suppresses acetylcholine-induced tentacular contractions. Animals were pretreated with 10 mM lidocaine for 20 min. Samples were then treated with 10 mM acetylcholine when indicated, showing loss of acetylcholine-induced tentacle contractions in *N. vectensis*. Red arrows highlight examples of animals that had lost tentacular contractions. The video was taken at 1× magnification and 7 frames per second.

**Additional file 15: Video S7.** Pretreatment with 10 mM Lidocaine suppresses nicotine-induced contractions. Animals were pretreated with 10 mM lidocaine for 20 min. Samples were then treated with 10 mM nicotine when indicated, showing loss of acetylcholine-induced tentacle contractions in *N. vectensis*. Red arrows highlight examples of animals that had lost tentacular contractions. The video was taken at 1× magnification and at 4 frames per second.

## Data Availability

For *N. vectensis* sequences, the JGI accession numbers are provided (Additional file 9: Table S2). Raw quantification, not shown within this paper, are available upon request.

## Abbreviations

nAChR: nicotinic acetylcholine receptor
mAChR: muscarinic acetylcholine receptor
hpf: hours postfertilization
dpf: days postfertilization
mpf: months postfertilization
