# Supplementary figures and images for: Characterization of nAChRs in Nematostella vectensis supports neuronal and non-neuronal roles in the cnidarian–bilaterian common ancestor
Source: EvoDevo. 2019 Nov 2;10:27. doi: 10.1186/s13227-019-0136-3 (PMC6825365; doi:10.1186/s13227-019-0136-3)

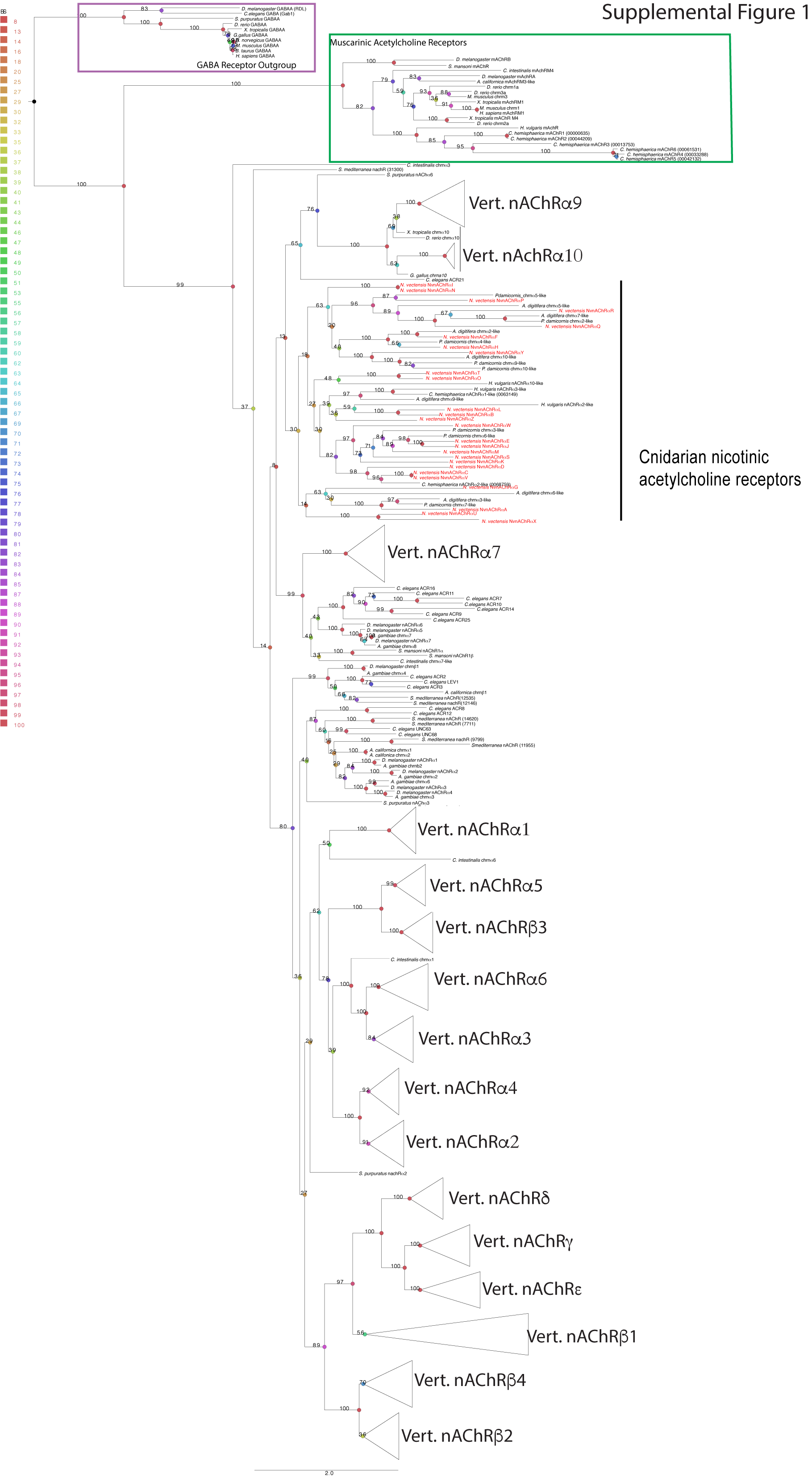

Supplement: Supplementary file 1 — Additional file 1: Figure S1. Phylogeny generated in RaxML using an unedited alignment. RaxML generated maximum likelihood phylogeny determining the relationship of potential cnidarian acetylcholine receptors to the known bilaterian nicotinic and muscarinic acetylcholine receptors. The GABA receptors, indicated by a purple box, served as the out-group for our analysis. Muscarinic receptors are indicated by a green box. The bootstrap values for critical nodes of interest are written in red. Tree generated using alignment in Additional file 9: Table S2. Heat map also indicates bootstrap values. N. vectensis sequences are in red text on tips of tree branches. [file 13227_2019_136_MOESM1_ESM.tif]

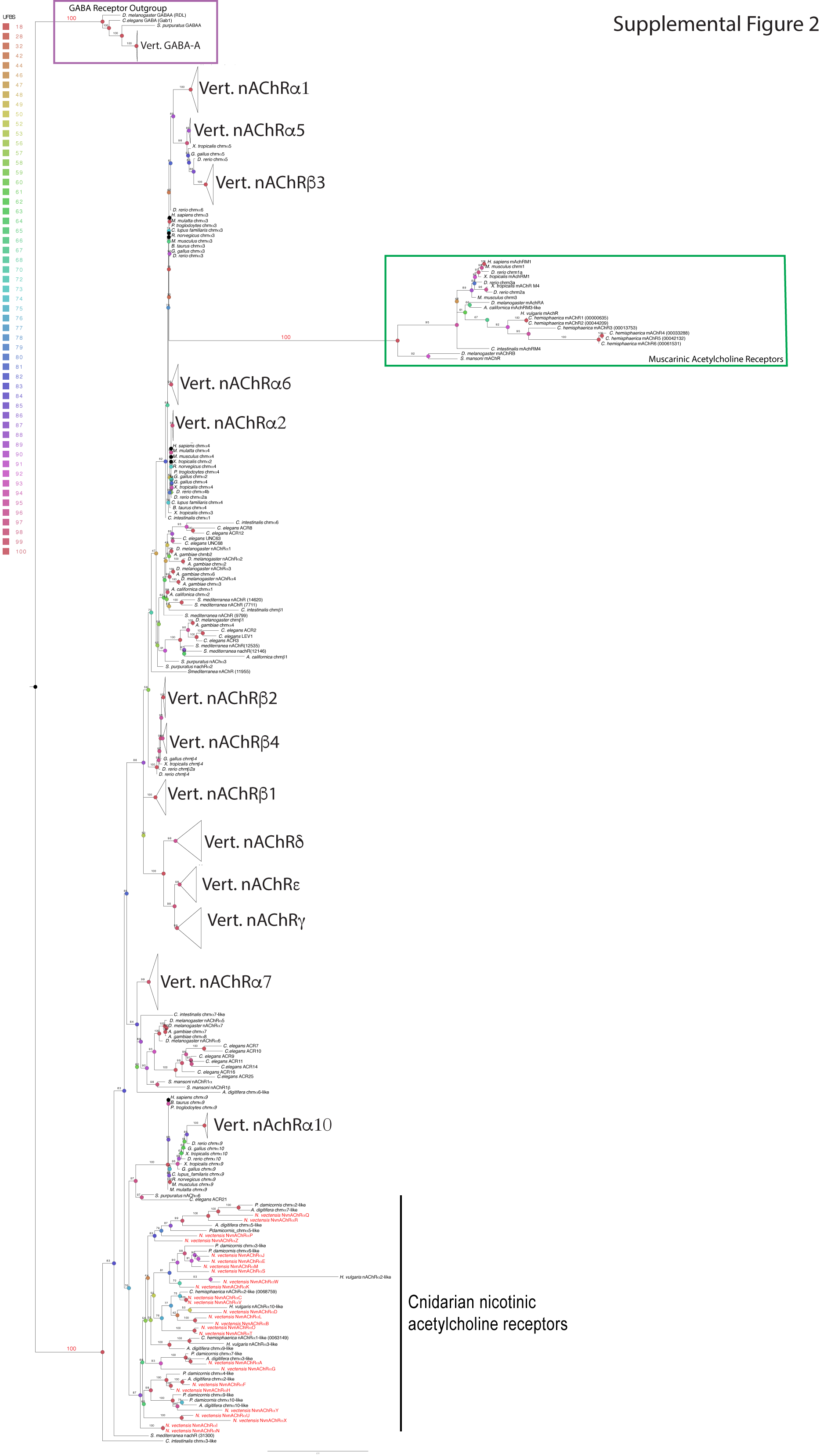

Supplement: Supplementary file 2 — Additional file 2: Figure S2. Phylogeny generated in IQ tree using an edited alignment that includes the transmembrane domains and double cysteines. IQ-tree generated maximum likelihood phylogeny determining the relationship of potential cnidarian acetylcholine receptors to the known bilaterian nicotinic and muscarinic acetylcholine receptors. The GABA receptors, indicated by a purple box, served as the out-group for our analysis. Muscarinic receptors are indicated by a green box. The ultrafast bootstrap values for critical nodes of interest are written in red. Tree generated using alignment in Additional file 10: Table S3. Heat map also indicates ultrafast bootstrap values. N. vectensis sequences are in red text on tips of tree branches. [file 13227_2019_136_MOESM2_ESM.tif]

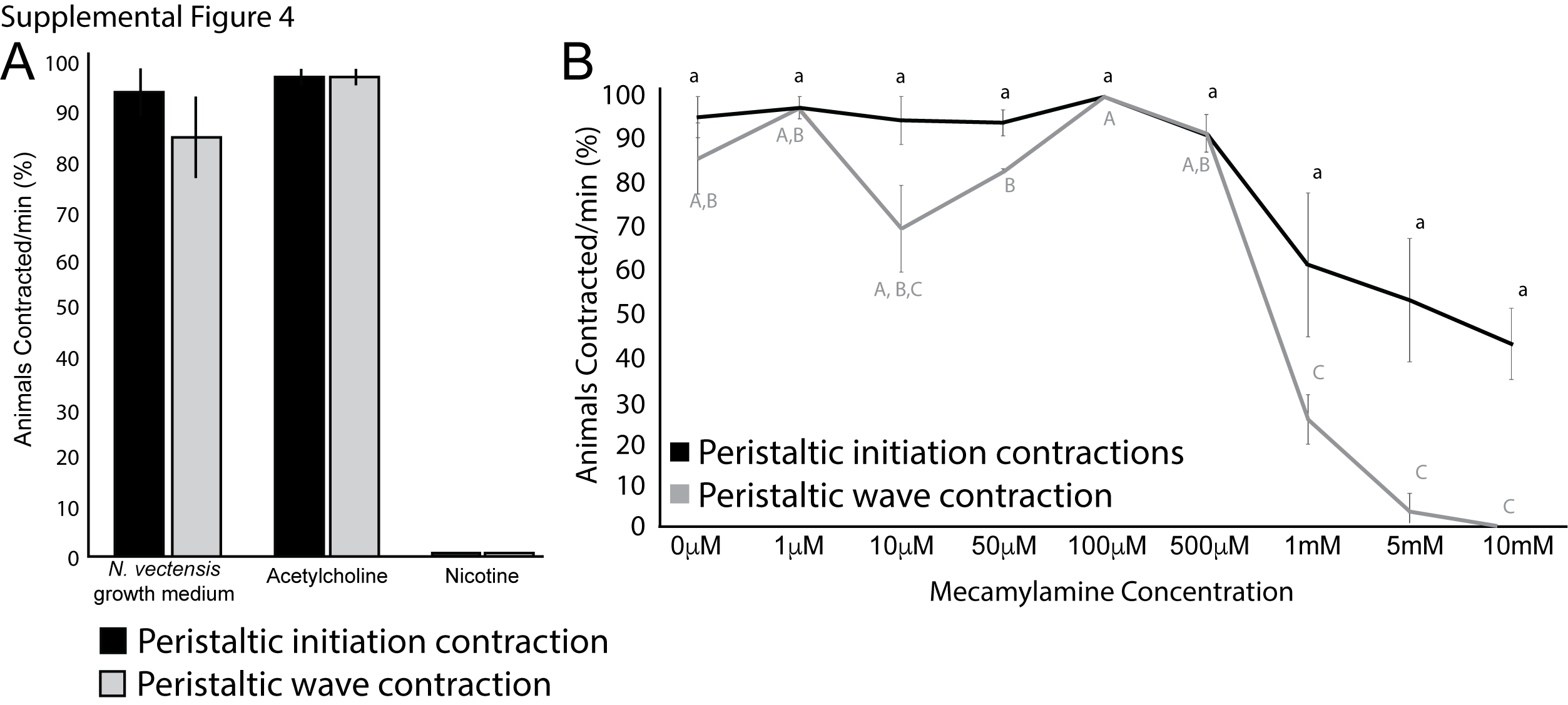

Supplement: Supplementary file 8 — Additional file 8: Figure S4. Quantifications of radial contractions and peristaltic waves in the presence of N. vectensis medium, acetylcholine, nicotine, and mecamylamine. (A) Treatment with acetylcholine did not induce a statistically significant difference in the number of animals with peristaltic initiation contractions (black) or peristaltic wave contractions (gray) (p ≥ 0.05). Treatment with nicotine alone resulted in complete radial contractions, thus no peristaltic wave contractions or peristaltic initiation contractions occurred. (B) Treatment with mecamylamine at 1 mM reduced the percentage of animals with peristaltic initiation contractions from ~ 91.53 ± 4.33% to 61.1 ± 16.6%. The peristaltic initiation contractions continued to drop to 42.6 ± 8.24% with 10 mM mecamylamine. A similar drop was observed in the percentage of animals who performed peristaltic wave contractions from 91.53 ± 4.33% at 500 µM to 25.39 ± 5.7% at 1 mM mecamylamine. The radial contractions were reduced to 0 ± 0% when treated with 10 mM mecamylamine. Each experiment was performed N > 3 times with an n ≥ 7/replicate. p values were calculated using a student t test for (A) and a one-way ANOVA for (B). (B, black) Peristaltic initiation contractions in 3-month-old polyps pretreated with 0 µM–10 mM mecamylamine F7,16 = 7.06, p < 0.005 (B, gray) Peristaltic wave contractions in 3-month-old polyps pretreated with 0 µM–10 mM mecamylamine F8,18 = 60.56, p < 0.001. Points that do not share letters, either uppercase or lowercase (B), are statistically different from each other. [file 13227_2019_136_MOESM8_ESM.tif]

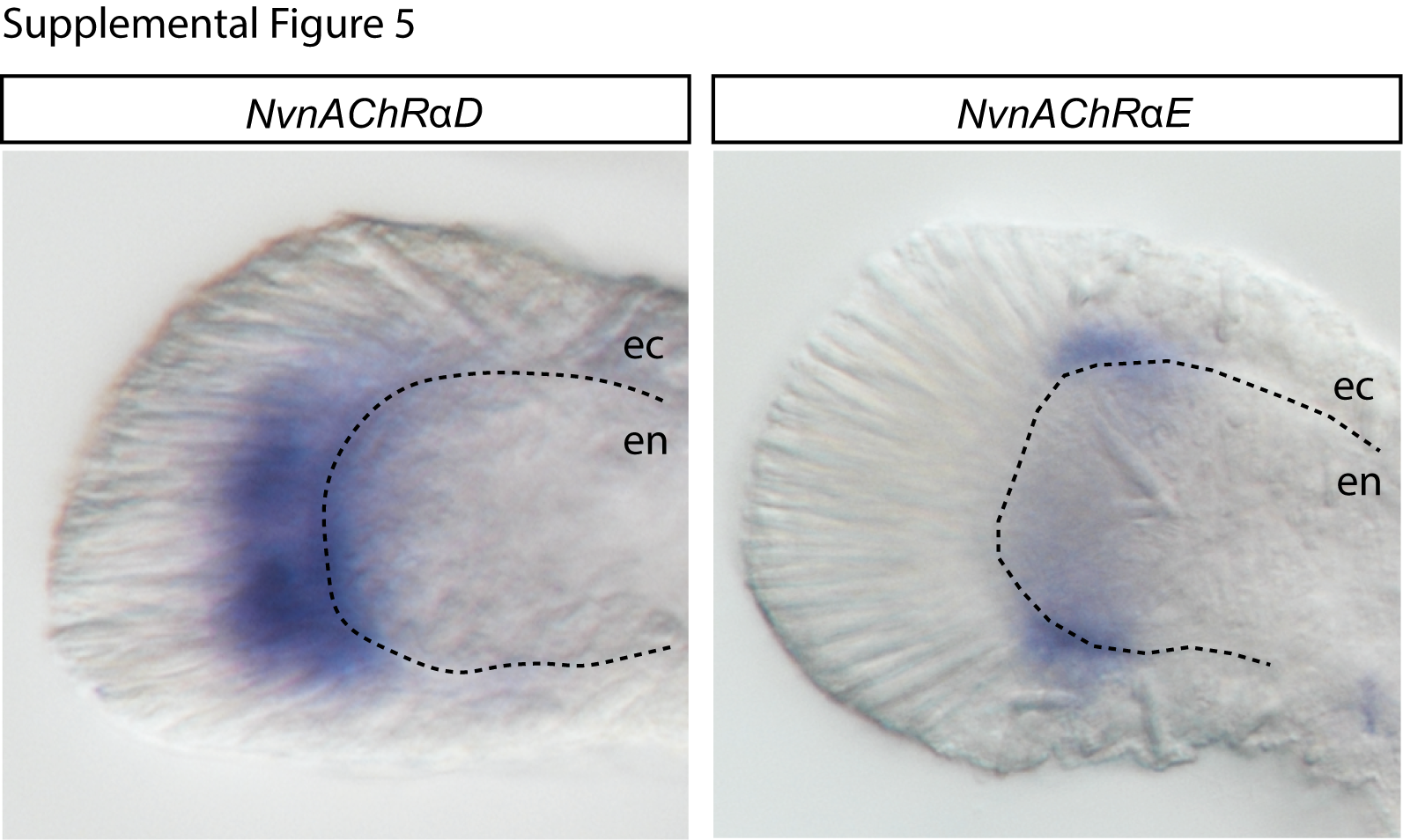

Supplement: Supplementary file 13 — Additional file 13: Figure S5. Expression of NvnAChRαD and NvnAChRαE in the tentacles. Expression of NvnAChRαD and NvnAChRαE are restricted to the tentacular ectoderm. Dotted line separates the ectoderm and endodermal tissue layers. ec = ectoderm, en = endoderm. [file 13227_2019_136_MOESM13_ESM.tif]
